# Emergency management of patients with Glanzmann thrombasthenia: consensus recommendations from the French reference center for inherited platelet disorders

**DOI:** 10.1186/s13023-023-02787-2

**Published:** 2023-06-29

**Authors:** Mathieu Fiore, Janine-Sophie Giraudet, Marie-Christine Alessi, Céline Falaise, Dominique Desprez, Roseline d’Oiron, Sophie Voisin, Marie-Françoise Hurtaud, Hélène Boutroux, Paul Saultier, Cécile Lavenu-Bombled, Gilles Bagou, Xavier Dubucs, Anthony Chauvin, Christophe Leroy, Francine Meckert, François Kerbaul, Nicolas Giraud, Ambra Pühler, Ana Rath

**Affiliations:** 1 Laboratoire d’hématologie, Centre de Référence des Pathologies Plaquettaires, CHU de Bordeaux, Hôpital Cardiologique, Inserm U1034 – Biologie des Maladies Cardio-Vasculaires, Pessac, France; 2 Centre de Référence des Pathologies Plaquettaires, Pessac, France; 3 ORPHANET, INSERM US14, Plateforme Maladies Rares, 96 Rue Didot, 75014 Paris, France; 4 Laboratory of Hematology, Aix Marseille Univ, APHM, INSERM, INRAe, C2VN, La Timone Hospital, Marseille, France; 5 Department of Pediatric Hematology, Immunology and Oncology, La Timone Children’s Hospital, Marseille, France; 6 Centre de Ressources et de Compétences des Maladies Hémorragiques Constitutionnelles, CHU de Strasbourg, Strasbourg, France; 7 Centre de Ressources et de Compétences des Maladies Hémorragiques Constitutionnelles, CHU du Kremlin-Bicêtre, Le-Kremlin-Bicêtre, France; 8 Laboratoire d’Hématologie, Centre de Référence des Pathologies Plaquettaires, CHU de Toulouse, Toulouse, France; 9 Laboratoire d’Hématologie, CHU Robert Debré, Paris, France; 10 Laboratoire d’Hématologie, Centre de Référence des pathologies Plaquettaires, CHU Armand Trousseau, Paris, France; 11 Department of pediatric hematology, immunology and oncology, Aix Marseille Univ, APHM, INSERM, INRAe, C2VN, La Timone Children’s Hospital, Marseille, France; 12 Anesthésiste-Réanimateur Urgentiste - SAMU-SMUR de Lyon - Hôpital Edouard-Herriot, 69437 Lyon Cedex 03, France; 13 Pôle Médecine-Urgences, CHU de Toulouse, Toulouse, France; 14 Président de la Commission des Référentiels de la SFMU (CREF), Chef de Service Adjoint - Service d’Accueil des Urgences/SMUR, CHU Lariboisière, Université de Paris, Paris, France; 15 Médecin Urgentiste - Service de Gestion des Crises Sanitaires - Département Qualité Gestion des Risques, Assistance Publique-Hôpitaux de Paris, Paris, France; 16 Direction Opérationnelle du Prélèvement et de la Greffe de l’Agence de la Biomédecine (ABM), Saint Denis, France; 17 Association Française des Hémophiles, Paris, France; 18 Reference Center of Platelet Disorders, APHM, Marseille, France; 19 Service Hématologie Biologique, Centre de ressources et compétences MHEMO, CHU Bicêtre, Assistance Publique-Hôpitaux de Paris, Faculté de médecine Paris Saclay, Le Kremlin-Bicetre, France

## Abstract

Glanzmann thrombasthenia (GT) is a genetic bleeding disorder characterised by severely reduced/absent platelet aggregation in response to multiple physiological agonists. The severity of bleeding in GT varies markedly, as does the emergency situations and complications encountered in patients. A number of emergency situations may occur in the context of GT, including spontaneous or provoked bleeding, such as surgery or childbirth. While general management principles apply in each of these settings, specific considerations are essential for the management of GT to avoid escalating minor bleeding events. These recommendations have been developed from a literature review and consensus from experts of the French Network for Inherited Platelet Disorders, the French Society of Emergency Medicine, representatives of patients’ associations, and Orphanet to aid decision making and optimise clinical care by non-GT expert health professionals who encounter emergency situations in patients with GT.

## Background

Glanzmann thrombasthenia (GT) is a rare autosomal recessive bleeding disorder caused by inherited defects of the platelet membrane α_IIb_β_3_ glycoprotein [1]. This receptor is required for platelet aggregation, which culminates in the formation of a ‘plug’ that closes the damaged site of the blood vessel [2]. Therefore, absence of platelet α_IIb_β_3_ glycoprotein prevents maintenance of vascular integrity in the case of injury leading to excessive bleeding [1, 3, 4]. GT is usually linked to quantitative, but sometimes qualitative, defects of the α_IIb_β_3_ complex [5]. The disease is classically divided into three subtypes: type I disease defined as less than 5% α_IIb_β_3_ expression; type II disease defined as residual α_IIb_β_3_ expression (5–20%); and variant disease defined as qualitative defect with at least 20% residual expression [6, 7]. In very rare cases, acquired GT is defined as spontaneous inhibition of normally expressed α_IIb_β_3_, which is most often associated with autoantibodies [8].

GT is usually suspected in cases with spontaneous or provoked mucocutaneous bleeding, such as epistaxis or heavy menstrual bleeding [9]. Symptoms may manifest shortly after birth, with purpura, umbilical stump haemorrhage and/or excessive bleeding following blood sampling for the Guthrie test. Nevertheless, in most patients, the diagnosis is made during childhood [7].

The laboratory criteria for diagnosis is based on the absence or severe decrease of platelet aggregation associated with reduced expression of α_IIb_β_3_ on the platelet surface detected by flow cytometry [1, 10]. However, clinical observations suggest little or no correlation between the amount of residual α_IIb_β_3_ expression and the severity of the disease [10].

Management of minor bleeding relies mostly on local haemostasis and/or the use of antifibrinolytics, whereas platelet transfusion remains the standard of care if bleeding cannot be controlled with these first measures or in cases of invasive procedures [11, 12]. Unfortunately, platelet transfusion therapy can be followed by an immune response that is directed against the deficient α_IIb_β_3_ complex and/or the HLA class I system [13]. These antiplatelet antibodies occur in approximately 20–30% of patients and they are of much clinical concern as they can render platelet transfusions ineffective [14]. In addition, data from animal models suggest that the hemostatic efficacy of transfused platelets is much lower in the absence of thrombocytopenia, as is the case in GT, because dysfunctional platelets can interfere with the hemostatic activity of transfused platelets [15]. Correspondingly, a large series of cases with congenital platelet disorders [16] confirms that the median platelet transfusion dose is significantly higher (median of 5) in patients without thrombocytopenia (such as GT patients). Nonetheless, administration of recombinant activated factor VII is a therapeutic alternative when platelet transfusions are ineffective or when platelets are not rapidly accessible [17].

As GT leads to major platelet functional defects, severe bleeding can occur suddenly, requiring prompt management by healthcare professionals who may not be aware of disease-specific features and related difficulties of care. The objective of these recommendations is to provide information on the best clinical practice in emergency situations that may arise in patients with GT based on data from the literature and the practical expertise of the French Reference Center for Inherited Platelet Disorders. These guidelines are intended to provide support in decision making for patient management and surveillance of possible complications.

## Methodology

Our working group identified a need for the development of recommendations for the management of GT patients in emergency situations, which are frequently encountered in this platelet disorder. Our aim was to make these recommendations easily accessible to medical practitioners working in emergency care on a daily basis. The elaboration of these standardised recommendations was coordinated by Orphanet [18] following a rigorous methodology. Since 2007, Orphanet has produced 112 recommendations for 447 different rare diseases [19], which are easily accessible through the websites of Orphanet or the French Society of Emergency Medicine and the Orphanet smartphone app. They also provide information for regulation by the emergency department in a single one-page table that can be read in 2–3 min by the emergency regulator and professionals sent to out-of-hospital sites.

This report combines the outcomes of a literature review and a national survey on physicians’ practises, and was elaborated in collaboration with members of the French Reference Center for Inherited Platelet Disorders, the French Society of Emergency Medicine, representatives of patients’ associations, and Orphanet. The production methodology of these recommendations was adapted from those elaborated by the French Authority for Health regarding good clinical practices. A prevalidated, standardised, and structured document was used as a draft, which included several different sections: *Basic principles*—these recommendations should be applicable by all emergency physicians regardless of the type of emergency department where the patient is receiving care; *Specific emergency situations*—divided into three different parts, i.e., emergency evaluation, immediate treatment and specific therapy; *Orientation*—recommendations regarding transport of the patient from home to the hospital, including where, how and when to transport; *Precautions regarding co-medication and vaccination*—discusses side effects or specific contraindications; *Anaesthesia-related risks and specific precautions*—specifies precautions related to tracheal intubation or other invasive procedures and anaesthetic drugs; and *Organ and tissue donation*—allows determination whether organ/tissue donation is possible based on current knowledge in collaboration with the French Biomedicine Agency.

All of these recommendations were validated by a panel of expert authors and proofreaders. In view of the rarity of the disease, the recommendations presented here are largely based on the daily clinical practise of the expert authors. Draft recommendations were circulated to the working group for comments and final approval. However, these guidelines are general in nature and as every patient is unique, only the attending physician can judge the suitability of their application in each specific situation.

Final recommendations are accessible in French within the Orphanet Emergency Guidelines collection and disseminated further through the Orphanet Guides app [20].

## Basic principles

It is imperative that health care professionals who may be unfamiliar with GT are made aware that GT patients are at high risk for bleeding [9]. Each patient or the parents/guardians of children with GT should possess a disease-specific emergency card as well as a logbook containing basic information on the disease and on GT-specific medical care [21]. Moreover, emergency practitioners should listen carefully to the patient, who will be intimately familiar his or her chronic disease. Some patients have undertaken therapeutic and educational programs regarding management of their disease [4].

Medical care should involve evaluation of the seriousness of the current situation based on clinical examination, and a specific protocol should be used for each patient in an emergency situation after asking for specialised advice from the referring centre involved in the patient’s ongoing care.

The use of a peripheral venous line (see also ‘Anaesthesia-related risks and specific precautions’ section) should be preferred and rectal temperature should not be taken.

Pre-transfusion screening blood tests should be performed and labile blood products in cooperation with the blood bank should be ordered, including in patients in clinically stable condition. It is recommended to monitor the evolution of bleeding as an indication for hospitalisation, and hospital discharge should be validated in consultation with an expert physician. Screening for antiplatelet antibodies should be performed on the day of admission and within 1–3 months following administration of blood products.

## Precautions regarding comedication and vaccination

Except in special cases, medications that can increase bleeding risk (nonsteroidal anti-inflammatory drugs, such as aspirin, and anticoagulants) should be avoided. In the case of pain, the use of paracetamol and major analgesics should be preferred [9].

Except in special cases, intramuscular administration should be avoided. Emergency vaccinations (e.g., tetanus immunisation) should be administered by subcutaneous injection into the deltoid region using a thin needle, followed by local and prolonged compression with a compressive bandage [4]. The use of tranexamic acid in cases with a recent thromboembolic event, severe renal impairment (risk of accumulation), or past history of seizures should be avoided.

## Specific emergency situations

### Spontaneous and provoked bleeding or haemorrhage

Emergency situations and specific appropriate therapies are described in Tables 1, 2 and 3. Hospitalisation is not mandatory in cases of minor bleeding.

**Table 1:** Specific emergency situations in Glanzmann thrombasthenia and their definition

Specific emergency situations	Definition
Life-threatening blood loss	Severe drop of hemoglobin level, hemorrhagic shock. For instance, high risk situations of bleeding complications: polytrauma—accidents in public road traffic (also in low kinetic state)—psoas muscle hematoma, deep wound—fracture—severe uterine bleeding particularly in case of menarche—hematemesis, melena—hemoptysis
Hemorrhage affecting functional prognosis	Intracerebral hemorrhage—eye injury—spinal hematoma—hematoma of the floor of the mouth—compartment syndrome …
Minor bleeding	Moderate epistaxis—gynecological hemorrhage—gum bleedings—sores in the mouth (frenulum, tongue or lips laceration) -loss of temporary teeth—tooth extraction—suturing of a wound—sprain—dislocation—moderate or mild muscular hematoma—hemarthrosis—hematuria—arterial puncture

**Table 2:** Details of emergency evaluation, immediate treatment and specific therapies that should be provided in case of severe bleeding in GT patients

Emergency evaluation	• Monitoring of vital parameters: blood pressure, heart and respiratory rates, oxygen saturation, temperature • Perform Whole Blood Count to evaluate the drop of hemoglobin level (platelet count is usually normal in GT) • Evaluate serum creatinine and ionogram • Perform routinely coagulation assay (results are expected to be normal in GT) • Pretransfusion blood screening test: blood group, rhesus, red cells phenotyping, detection of irregular agglutinins, anti-HLA and anti-α_IIb_β_3_ antibodies (without waiting for results)
Immediate treatment	• Use local hemostatic means (compression if possible) and cryotherapy (15–20 min. every 6 h) • Give analgesics (avoid administration of non-steroidal anti-inflammatory drugs) • Give antibrinolytics by systemic route (1 g and 10 mg/kg every 8 h in adult patients and children, respectively) • Red blood cells administration if required
Specific therapies	• Ask for expert medical advice and discuss:
	Platelet transfusion (HLA-matched concentrates if necessary)
	rFVIIa in case of refractoriness to platelet transfusion or when platelets are not readily available (90 ug/kg every 2 h; at least 3 doses should be administered before concluding that the therapy has failed)

**Table 3:** Recommended therapies in different and specific clinical situations

Specific emergency situations	Recommendations
Skin injury	• Prolonged compression of the skin lesion (at least 10 min) using compresses eventually soaked with tranexamic acid • Antiseptic solutions, excluding alcool products • Eventually use a hemostatic and compressive bandage
Epistaxis	• Reassure the patient • Place the patient in a semi seated position with the head bent forward • Ensure of the absence of posterior bleeding by examination of the throat, especially in children • Be aware of the possibility of blood ingestion or inhalation mimicking gastrointestinal bleeding or hemoptysis with respiratory distress • Blow the nose to evacuate blood clots and limit local fibrinolysis • Nasal compression maintained at least 10 min using both fingers • Apply cold (ice pack) if necessary • Give oral tranexamic acid during 7 to 10 days to avoid recurrence of bleeding *In case of failure:* • Bilateral packing anterior to the septum using hemostatic and absorbable compresses eventually soaked with tranexamic acid • Antibiotherapy is required during all the period of packing • Patient monitoring should be planned with an ENT specialist • If bleeding persists, the use of a balloon or a packing posterior to the septum will be discussed
Loss of temporary teeth or gum bleeding	• Prolonged compression of the gum and application of a hemostatic tissue sealant if necessary • Use oral antifibrinolytics during 10 days • Mandatory monitoring to adapt the treatment in case of failure • If bleeding persists, patient should see a dentist
Heavy menstrual bleedings	• Evaluate the severity of bleeding (drop of hemoglobin level) • Give oral tranexamic acid • The use of NSAIDs should be avoided • Consider the possible need for hospitalization in case of major bleeding that could require blood transfusions • Gynecological assessement is required: discuss the use of hormonal therapy with a monophasic pill containing at least 30 µg of ethinylestradiol • Treatment of iron deficiency

### Emergency surgery

GT patients should be treated in a medical centre with availability of follow-up 24 h per day and easy access to blood products [12]. It is highly recommended to contact the referring centre involved in the patient’s ongoing care to define appropriate management [21].

Management must be supported by a multidisciplinary team (surgeon, anaesthetist, haematologist, pharmacist, and provider of blood products) after evaluating the patient’s bleeding history (spontaneous or provoked haemorrhagic syndrome, blood transfusions, and antiplatelet antibodies), results of urgent blood sampling, and type of surgery [22–24]. If possible, a written protocol should be established with details regarding the recommended haemostatic treatment, anaesthesia-related risks, and precautions that should be taken (see ‘Anaesthesia-related risks and specific precautions’ section) [25, 26]. Contact numbers of the medical team and referring specialists should also be included in the protocol [27]. After having taken into account the bleeding risk of surgery and that patient’s platelets may interfere with transfused ones, platelet concentrates should be administered 1 h before surgery and then every 12–24 h [16, 24]. In cases refractory to platelet transfusion or when platelets are not readily available, rFVIIa should be administered 10 min before surgery and then every 2–3 h [17, 23, 26]. Caution should be taken regarding the risk for thrombosis when using rFVIIa, and thrombosis prophylaxis should be decided on an individual basis [28–30]. Administration of intravenous or oral tranexamic acid may also be used [22]. Measures to improve local haemostasis, such as surgical haemostasis, surgical approaches, and local application of tranexamic acid or bioadhesives, should be applied. Perioperative blood loss must be evaluated regularly. Postoperative monitoring should be continued as long as the bleeding risk persists. Therefore, ambulatory care will be limited to conditions with a minor bleeding risk (e.g., dental care, cataract surgery, minor skin surgery).

### Emergency delivery

Delivery should be in a maternity clinic capable of an adequate level of support [31]. The choice of delivery mode should first be determined according to obstetric conditions [32]. Caesarean section may, in some cases, be preferable for organisational reasons depending on the availability of blood products and local coordination [33]. The presence of antiplatelet antibodies should also be screened without waiting for final results [34, 35]. Choice of therapy should take into account recent or past history of platelet transfusion refractoriness. In the absence of a previously established medical care protocol, management should be similar between vaginal delivery and caesarean section, relying on the use of platelet concentrates and/or rFVIIa with or without tranexamic acid. The use of surgical drains in cases of caesarean section and regular monitoring of haemoglobin rates during the first 12–24 h will allow rapid detection of abnormal bleeding. Systematic assistance with delivery of the placenta is also recommended to limit blood loss and prevent postpartum haemorrhage [27, 33, 36]. The duration of therapy will depend on the clinical evolution. As GT is an autosomal recessive disorder, in the absence of consanguinity, the neonate will be an obligate asymptomatic heterozygous carrier [7]. However, the infant may be affected by neonatal immune thrombocytopaenia induced by the transplacental passage of maternal anti-α_IIb_β_3_ antibodies that may require specific interventions [37, 38].

## Anaesthesia-related risks and specific precautions

Bleeding risk before any invasive procedure should be evaluated carefully [16, 39] and any invasive procedures should be performed by a qualified practitioner [27]. Epidural or spinal anaesthesia, locoregional anaesthesia, and analgesics administered via the intramuscular route should be strictly avoided [27, 40]. If tracheal intubation or catheterisation is required, sedation will be necessary to limit traumatic injuries. Due to the bleeding risk during their placement or removal, the use of central venous lines should be avoided whenever possible (subclavian or femoral vein access). Central venous line insertion should be performed using ultrasound-guided access and after haemostatic correction. In cases of ophthalmic surgery, retrobulbar block is contraindicated and topical anaesthetics, such as eye drops, should be used [41, 42].

## Orientation

### Transport from home to the emergency department

#### Where to transport?

Sometimes it may be necessary for the emergency medical dispatcher to guide the ambulance to an appropriate destination hospital according to bleeding risk and the type of emergency department available (e.g., emergency department for adults or children, intensive/critical care unit). This will be balanced by emergency management and the priority to guide the patient toward an institution where technical facilities and expertise are readily accessible. The patient will be admitted directly to the emergency department after prior agreement between practitioners in a medical centre where follow-up and ready access to blood products 24 h per day are possible. Expert medical advice from the referring practitioner should always be sought.

#### How to transport?

The emergency medical dispatcher may help to define the mode of medical transport with or without a fully equipped ambulance depending on the clinical situation. The type of transport (land or helicopter) will depend on where the patient is located (accessibility, distance to the healthcare centre) and the severity of the clinical situation.

#### When to transport?

The patient should be transported immediately in the case of life-threatening blood loss.

### Orientation following management by the emergency department

#### Where to transport?

The patient should be transported to the department of medicine/surgery or intensive/critical care unit depending on the severity of the clinical situation.

#### How to transport?

A fully equipped ambulance may or may not be required depending on the clinical situation.

#### When to transport?

The patient should be transported once the clinical situation has stabilised. Moreover, the patient should not leave the emergency department until expert advice has been obtained.

## Organ and tissue donation

Current knowledge suggests that postmortem organ and tissue donation are possible depending on assessment of each patient’s situation. The referring centre or local biomedical agency should be contacted. These recommendations are based on those established by the French Biomedical Agency.

### Risk of disease transmission

The disease can be transmitted by bone marrow donation, which is contraindicated.

### Specific risk related to the disease or medical treatment received

There is a risk of transmission of viral infections linked to the history of blood product administration, but only HIV infection uncontrolled at the time of the donation is currently considered an absolute contraindication. Organ donation in patients with hepatitis B or C virus infection, even if currently active, is permitted under certain conditions. The decision regarding organ transplantation will be based on the risk incurred by the recipient compared to the expected benefit.

### Organ donation

Organ donation is possible subject to the clinical and paraclinical evaluation of the donor, the organ, and treatment followed.

### Tissue donation

Subject to individual evaluation, tissue donations (cornea, blood vessels, cardiac valves, skin, bone, etc.) are possible.

## Conclusion

**Fig. 1:**
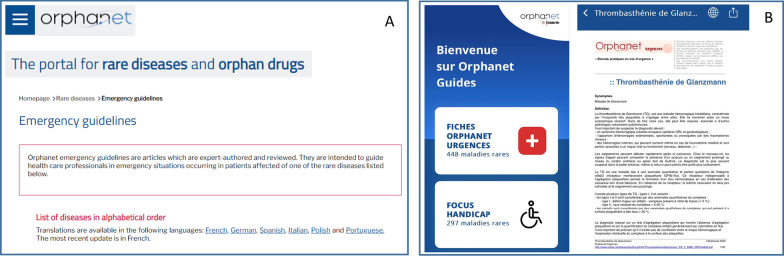
Screenshots of Orphanet website (**A**) and the French application of Orphanet for mobile phone (**B**) where emergency recommendations are easily accessible

Different medical emergency situations and related complications can occur in GT patients due to the high risk of bleeding associated with this severe, inherited platelet disorder. Specifically, polytrauma, deep wounds, or severe uterine bleeding may result in life-threatening blood loss, whereas minor trauma can cause moderate bleeding that may worsen over time. These clinical situations require prompt treatment to reduce the period of required clinical management. Although acute management of these emergencies should follow the same basic principles as in non-GT patients, specific care should be taken to correct conditions unique to this disorder. The recommendations provided here should help nonspecialist physicians to safely and appropriately manage GT patients until expert advice can be obtained from the referring team [20]. Such expert advice can be obtained easily through the websites of Orphanet (15 million users per year) or the French Society of Emergency Medicine and the smartphone Orphanet app (possible use at patients’ bedside) (Fig. 1).

## Data Availability

Data sharing is not applicable to this article as no datasets were generated or analysed during the current study.

## Abbreviations

GT: Glanzmann thrombasthenia
